# Transport Reversal during Heteroexchange: A Kinetic Study

**DOI:** 10.1155/2013/683256

**Published:** 2013-10-26

**Authors:** V. Makarov, L. Kucheryavykh, Y. Kucheryavykh, A. Rivera, M. J. Eaton, S. N. Skatchkov, M. Inyushin

**Affiliations:** ^1^Department of Physics, UPR, San Juan, PR 00931, USA; ^2^Department of Biochemistry, UCC, Bayamon, PR 00960, USA; ^3^Department of Physiology, UCC, Bayamon, PR 00960, USA

## Abstract

It is known that secondary transporters, which utilize transmembrane ionic gradients to drive their substrates up a concentration gradient, can reverse the uptake and instead release their substrates. Unfortunately, the *Michaelis-Menten* kinetic scheme, which is popular in transporter studies, does not include transporter reversal, and it completely neglects the possibility of equilibrium between the substrate concentrations on both sides of the membrane. We have developed a complex two-substrate kinetic model that includes transport reversal. This model allows us to construct analytical formulas allowing the calculation of a “heteroexchange” and “transacceleration” using standard Michaelis coefficients for respective substrates. This approach can help to understand how glial and other cells accumulate substrates without synthesis and are able to release such substrates and gliotransmitters.

## 1. Introduction

Unlike “primary” or ATP dependent transporters that create the major ionic gradients of K/Na/H and Cl/CO_2_ ions across cellular membranes harnessing the energy reserved in ATP, the “secondary transporters” utilize the energy available from transmembrane ionic and/or pH gradients and membrane potential to drive their substrates up a steep concentration gradient. Transporters on neurons and astrocytes clearing neurotransmitters from the synaptic cleft and extracellular space mainly belong to different “secondary transporters” families. Recently, it has been shown that astrocytes and other glial cells accumulate monoamines [1] and polyamines [2, 3] while lacking the enzymes for their synthesis [1, 4–6]. One among many known representatives of the “secondary transporters” that utilize the transmembrane ionic gradients and membrane potential is the family of organic cation transporters (OCT). These transporters take up different mono- and polyamines [7], and cells expressing such transporters also release these substrates using possibly two pathways: (i) large pores and (ii) transport reversal. Here we analyze one of transport reversal mechanisms. 

*Energy Calculations*. Experimentally, it has been shown that secondary transporters can reverse their uptake releasing their substrates instead [8–10]. Energy based calculations were introduced to analyze the conditions for substrate release or uptake for this kind of transporter [11, 12]. It was established that substrate transport depends on the energy balance of coupled transport of the substrate and simultaneously transported ions (see Appendix A). Most secondary transporters could be reversed by membrane potential and by changes in the principal ion gradients and substrate concentrations. Experimentally, the reversal was shown for the glutamate transporters (for the review see [13]), GABA (reviewed by [12]), and for glial organic cation transporters [14, 15]. Being reversed, electrogenic transporters usually change the direction of the net ion flow. We summarize the energy balance study, introduced by Rudnick [11] in Appendix A. This analysis only studies one substrate uptake/release by a secondary transporter. 

*Michaelis-Menten Scheme*. The kinetic concept based on the Michaelis-Menten scheme proved very useful for transporter mediated substrate uptake and inhibition [16, 17]. This kinetic model predicts saturability and specificity of secondary transporters in many cases, and atypical transport kinetics can be explained by multiple binding sites [18]. We have summarized this classic concept in Appendix B. Unfortunately, as one can see, the Michaelis-Menten model does not include transporter reversal, and it completely neglects the reversal constant (see Appendix B). A more complex transporter kinetic model is needed to predict quantitatively at least the following well-established experimental observations.

1. It has been shown that one transporter substrate can release another one already accumulated inside the cell. Sometimes this is called “heteroexchange.” For example, dopamine, tyramine, and amphetamine, which are substrates for the neuronal dopamine transporter (DAT), can release the substrate named N-methyl-4-phenylpyridinium (MPP) through DAT [19], with releasing ability of these substances correlated with the elicited coupled transport current. Also, it was shown that L-glutamate and its transportable analogs (substrates for EAATs) specifically release L-aspartate (another EAAT substrate) through this transporter and can be blocked by nontransportable analogs [20].
2. A special term was coined for the release of the (tracer) substrate by the same substrate, a process named “transacceleration.” While the phenomenon is not kinetically different from the “heteroexchange” described in the previous paragraph, it is well established experimentally (see, e.g., [21]). As new transporter models arise (e.g., a channel-transporter model [22]), it might be important to get this phenomenon explained by a purely thermodynamic model, not by using kinematic assumptions.

Here we present kinetic algorithms that more accurately explain the behavior of a secondary transporter pumping two substrates simultaneously; it predicts transporter reversal by the application of an additional second substrate to the transporter already in equilibrium with the first substrate, “transacceleration” and other interactions. 

## 2. Results

We modified the Michaelis-Menten kinetic model to include two different transportable substrates and also additional elementary steps, characterized by their kinetic coefficients, which are necessary for the transporter not only to uptake but also to release substrates. The model is presented in Appendix C by relations 1–8. This model can be considered as a system of kinetic equations describing the dynamics of the model ((C.2))–((C.11)). A general solution for this scheme is difficult to obtain analytically. But some particularly interesting cases can be resolved (see Appendices C.1, C.2, and C.3), and we are presenting them below.

### 2.1. Equilibrium Conditions for Both Substrates (See Appendix C.1)

Practically, the initial concentrations of substrates *S*_1_ and *S*_2_ are considered known (i.e., *S*_10_ and *S*_20_), and then substrate concentration can be measured in the outside solution (*x*_1_ and *x*_2_ in our notation for this section of Appendix C). In this way, we tried to reduce all equations to measurable parameters.

**Figure 1:**
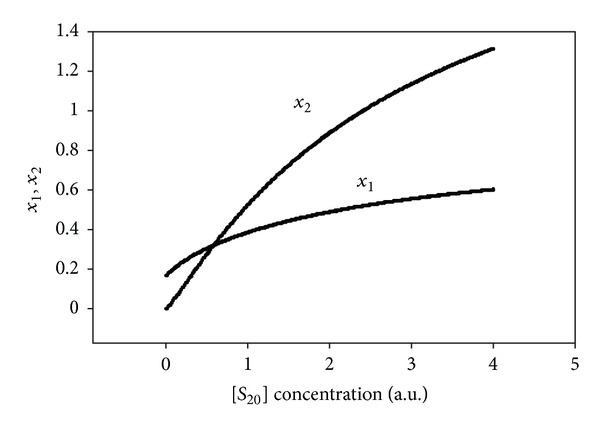
Dependence of *x*_1_, *x*_2_ on *S*_20_ at *S*_10_ = 1.5 a.u., *K*_11_ = 1.5, *K*_12_ = 1.8,  *K*_21_ = 1.3, *K*_22_ = 2.

It follows from relationships ((C.30)) (Appendix C) that at fixed concentration of *S*_10_ (initial concentration of first substrate *S*_1_) and variable concentration of *S*_20_ (different initial concentrations of *S*_2_), the equilibrium concentration of *x*_1_ (the *S*_1_ substrate outside) increases with increasing *S*_20_ (the effect of *S*_1_ substrate *releasing* from the cell), and similarly, the equilibrium concentration of *x*_1_ decreases with decreasing *S*_20_ (effect of *S*_1_ substrate transport inside of the cell). The same behavior follows from ((C.30)) for the equilibrium concentration of *x*_2_ at fixed concentration of *S*_20_ and variable concentration of *S*_10_. These respective dependencies are shown in Figure 1. 

*Conclusions of Appendix C.1*

(1) Effect of substrate being *released* in case of competition in the two-substrate system can be observed, if at equilibrium condition most of the transporter is coupled by both substrates of interest: *T*_0_ ≫ *y*.
(2) Efficiency of the substrate *releasing* process is dependent on equilibrium constant values describing processes of substrate-transporter intermediate complex formation.
(3) Correct sign of the square root term in relations ((C.30)) is defined by the conditions of
(1) $$\begin{array}{c} S_{10}\geq x_{1}\geq 0,\\ S_{20}\geq x_{2}\geq 0. \end{array}$$
(4) Relationships ((C.30)) can be used for analysis of equilibrium substrate concentration dependence on initial substrate concentrations.
(5) The relationship of
(2) $$\begin{array}{c} x_{2}=\frac{S_{10}+S_{20}-\alpha x_{1}}{\beta } \end{array}$$
  can be used to determine parameters *α* and *β*, if concentrations of *x*_1_ and *x*_2_ can be simultaneously measured as functions of *S*_10_ and *S*_20_, the initial concentrations of the first and second substrates.

### 2.2. Two-Substrate System Dynamics at the Initial Time

Transporter velocities (transport rates) can be determined if (similar to Michaelis-Menten scheme) there is no equilibrium between substrate concentrations inside and outside and processes of the type

(3) $$\begin{array}{c} S_{1}^{\prime }\;+\;T\;\to \;\left(S_{1}T\right),\\ S_{2}^{\prime }\;+\;T\;\to \;\left(S_{2}T\right) \end{array}$$

can be neglected. We also assume there are the initial conditions where *S*_1_ and *S*_2_ are added to the external solution, thus *x*_1_ = *S*_10_ and *x*_2_ = *S*_20_. In that case (see Appendix C.2),

(1)
(4) $$\begin{array}{c} \upsilon_{x1}=k_{12}K_{1}\frac{S_{10}\left[T_{0}\right]}{1+K_{1}S_{10}+K_{2}S_{20}}, \end{array}$$
(5) $$\begin{array}{c} \upsilon_{x2}=k_{22}K_{2}\frac{S_{20}\left[T_{0}\right]}{1+K_{1}S_{10}+K_{2}S_{20}} \end{array}$$
  are analogous to the Michaelis-Menten formulation for a two-substrate system. If *S*_20_ = 0, we obtain the exact Michaelis-Menten formula for the first substrate velocity, and if *S*_10_ = 0, we obtain the exact Michaelis-Menten formula for the second substrate velocity. Note also that the term *k*_12_[*T*_0_] can be interpreted as *V*_1max_, and *k*_22_[*T*_0_] as *V*_2max_.
(2) The constants *k*_12_, *K*_*M*1_, *k*_22_, and *K*_*M*2_ can be determined experimentally similar to the standard procedures used in the Michaelis formulation. There are some important equations:
  (i) if *S*_10_ ≫ *K*_*M*,2_ and *S*_10_ ≫ *αS*_20_:
(6) $$\begin{array}{c} \upsilon_{1,s}=k_{12}\left[T_{0}\right],\;\text{the velocity at maximum,}\;\;V_{\text{1max}},\\ \upsilon_{x2}=k_{22}\frac{\alpha S_{20}\left[T_{0}\right]}{S_{10}}. \end{array}$$
  (ii) If *S*_10_ ≪ *αS*_20_ and *αS*_20_ ≫ *K*_*M*,1_:
(7) υx1′=dx1dt=k12S10[T0]αS20,υ2,s′=k22[T0], the velocity at maximum,  V2maxυ1,sυ2,s=k12S10[T0]αS20k22αS20[T0]S10=k12k22[T0]2  (see  Appendix  C).

### 2.3. Effect of the Equilibrium Reverse Bias for a First Substrate When a Second One is Added to the System

If previously the equilibrium was established for a first substrate between outside concentration of the substrate and the inside concentration, the addition of a second substrate will produce a reverse bias (equilibrium shift). In the beginning, at initial time, some of the transporter molecules in the outside bind to the second substrate while inside there is still no second substrate. That means the availability of outside transporter for a first substrate becomes reduced. Thus equilibrium for a first substrate starts to break down; that is, the velocity of first substrate transport to outside (release) becomes bigger than its transport to the inside. At initial times during the start of the process and far from equilibrium for a second transporter, (8) allows the calculation of the velocity of the first substrate release due to transport reversal (see Appendix C.3):

(8) $$\begin{array}{c} \upsilon_{x1}=A\left[1-\frac{K_{M,2}}{S_{20}+K_{M,2}}\right], \end{array}$$

where *K*_*M*,2_ is Michaelis constant for a second substrate and *S*_20_ is initial concentration of a second substrate,

(9) $$\begin{array}{c} A=\frac{k_{11}^{\prime }S_{10}}{K_{0}+1}y_{0},\\ y_{0}=\frac{\left[T_{0}\right]\left(K_{11}K_{12}+1\right)}{K_{11}S_{10}+K_{11}K_{12}+1}=\frac{\left[T_{0}\right]}{K_{S}S_{10}+1}, \end{array}$$

where

(10) $$\begin{array}{c} K_{S}=\frac{K_{11}}{\left(K_{11}K_{12}+1\right)}. \end{array}$$

Thus, finally we have

(11) $$\begin{array}{c} A=\frac{k_{11}^{\prime }S_{10}}{K_{0}+1}\frac{\left[T_{0}\right]}{K_{S}S_{10}+1}. \end{array}$$

**Figure 2:**
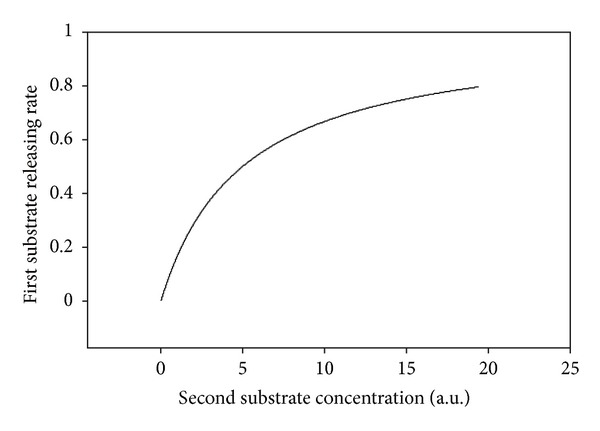


Taking into consideration the relation (8) we have calculated the dependence of the velocity of first substrate release on the concentration of a second substrate, at initial times after it was added to the system. Functional dependence (8) is represented in Figure 2.

It can be seen from (8) and Figure 2 that the velocity of reversed transport (release) of a first substrate is 0 if *S*_20_ = 0, because there is equilibrium between the velocities of inward and outward flow of the first substrate through the transporter. Thus, the “net” velocity, is equal to zero. Also, from (8) and as seen in Figure 2, with increase of a second substrate concentration, when *S*_20_ ≫ *K*_*M*,2_, the velocity of a first substrate release becomes saturated and can be calculated as

(12) $$\begin{array}{c} \upsilon_{x1,S}=A=\frac{k_{11}^{\prime }S_{10}}{K_{0}+1}\frac{\left[T_{0}\right]}{K_{S}S_{10}+1}. \end{array}$$

Release velocity depends on the first substrate concentration *S*_10_, and at given value of *S*_10_ the value of *A* is a constant. Thus, (8) for the velocity at half maximal value at a certain concentration of second substrate *S*_20_ can be written as

(13) $$\begin{array}{c} \upsilon_{x1}=A\left[1-\frac{K_{M,2}}{S_{20,1/2}+K_{M,2}}\right]=\frac{A}{2}, \end{array}$$

and because of this equation it can be calculated as

(14) $$\begin{array}{c} K_{M,2}=S_{20,1/2}. \end{array}$$

There is similarity between the formula of velocity of transporter reversal due to second substrate addition and Michaelis-like formulas for the velocity of substrate uptake. 

The formula that predicts the velocity of substrate uptake (see Appendix C ((C.54)) or Appendix B ((B.10))) can be written as

(15) $$\begin{array}{c} \upsilon_{x2}=\frac{k_{22}S_{20}\left[T_{0}\right]}{S_{20}+K_{M,2}}, \end{array}$$

where the maximum velocity is represented by

(16) $$\begin{array}{c} \upsilon_{x2,\max}=k_{22}\left[T_{0}\right]. \end{array}$$

Thus, for the half maximal velocity,

(17) $$\begin{array}{c} \frac{\upsilon_{x2,\max}}{2}=\frac{k_{22}S_{20,1/2}\left[T_{0}\right]}{S_{20,1/2}+K_{M,2}}=\frac{k_{22}\left[T_{0}\right]}{2}. \end{array}$$

Thus we can write

(18) $$\begin{array}{c} K_{M,2}=S_{20,1/2}. \end{array}$$

To say in plain words, the Michaelis constant for a second substrate can be determined in two ways: (i) from the standard Michaelis formulas at transport velocity measurements for the second substrate, or (ii) from the release velocity measurements of a first substrate, from our formula, where a second substrate produces release of the first one. 

In the most important case, if *K*_*S*_ *S*_10_ ≫ 1  (8)

(19) $$\begin{array}{c} A_{S}=\frac{k_{11}^{\prime }}{K_{0}+1}\frac{\left[T_{0}\right]}{K_{S}}=\text{Const}.,\\ \upsilon_{x1,S01}=A_{S}\left[1-\frac{K_{M,2}}{S_{20}+K_{M,2}}\right], \end{array}$$

then *A*_*S*_ can be interpreted as the release force for a first substrate after the addition of a second one. In the case of *K*_*S*_ *S*_10_ ≪ 1, the release force can be written as

(20) $$\begin{array}{c} A\left(S_{10}\right)\approx A_{S}S_{10}=\frac{k_{11}^{\prime }}{K_{0}+1}\frac{\left[T_{0}\right]}{K_{S}}S_{10}; \end{array}$$

that is, in this case the release force for a first substrate after the addition of a second one has a linear dependence on the first substrate concentration.

## 3. Discussion and Conclusions

We have studied the extended kinetic model for a secondary transporter simultaneously dealing with two substrates, which includes direct (outside-in) and reverse transport (inside-out). The model was solved in different equilibrium conditions (see Appendices C.1, C.2, and C.3). We have shown that when both substrates are in equilibrium, addition of one of them leads to reequilibrium and release of the second substrate (Appendix C.1). This was emphasized in Appendix C.3, when the system was studied for conditions where a first substrate is in equilibrium (inside-outside concentrations) and a second one is just added and is far from equilibrium. This situation is of a special interest as it has been studied experimentally [19, 20]. Also, this is what probably happens when methamphetamine, ephedrine, or other similar substances induce dopamine (and other monoamine) release from monoamine neurons primarily via membrane transporters, reversing the dopamine transporter (DAT), norepinephrine transporter (NET), and/or serotonin transporter (SERT) [23–27] and also reversing VMAT vesicular transport [28]. In addition, it has been recently shown that astrocytes and other glial cells accumulate polyamines [2, 3] while lacking the enzymes for their synthesis [4–6], and OCT type of transporters (that are expressed in glia) take up different polyamines [7]. Polyamines are released in brain from glial cells, but the mechanisms of such release are unknown [29].

Actually, as we understand now from formula (8) it can be ANY transportable substrate. This formula allows us to classify experimental measurements of a “heteroexchange” related substrate release for substrate-transporter pairs, using standard Michaelis coefficients.

The special term for the release of the (tracer) substrate by the same substrate, a process named “transacceleration,” can be explained by changes in equilibrium according to formula (8). There is no fundamental thermodynamic difference if the system has two chemically distinct substrates for the same transporter or there are radiolabelled and unlabelled chemically similar substrates. Thus, a new added substrate produces the release of a similar tracer substrate (labelled, e.g., with radioactive isotope) by equilibrium shift as shown in Appendix C.3. 

 We also have shown that if we assume both substrates are far away from equilibrium, and transporter reversal can be neglected (Appendix C.2), the formulas for the uptake velocity of both substrates become the same as in the Michaelis-Menten scheme (see Appendix C.2, ((C.53)) and ((C.54))), with the respective inhibitory coefficients.

We suggest that formula (8) will be especially useful in the study of polyspecific transporters with known multiple substrates, such as the organic cation transporters (OCT) that participate in the transport of different monoamines [30], as well as polyamines [7].

## Appendices

### A. Classical Energy Based Calculations

Similar to the analysis of ion channels, substrate flux through transporters can be determined by the transmembrane electrochemical potential ( $\Delta \tilde{\mu }$ ) which is the sum of the electrical potential (ΔΨ) and chemical potential (Δ*G*). For a single molecule *X*, the driving force is quantified as

(A.1)
$$\begin{array}{c} \Delta {\tilde{\mu }}_{x}=\Delta \Psi_{x}+\Delta G_{x}=z_{x}\cdot F\cdot E_{m}+RT\cdot \ln\frac{{\left[X\right]}_{\text{in}}}{{\left[X\right]}_{\text{out}}}, \end{array}$$

where *z*_*x*_ = the valence of *X*, *F* = Faraday's constant, *E*_*m*_ = membrane potential, *R* = universal gas constant, and *T* = temperature. It should be noted that when $\Delta \tilde{\mu }=0$ (i.e., if *X* is at equilibrium), this equation reduces to the Nernst equation.

For the glial glutamate transporter GLT1, for example, all of the cotransported ions are coupled to each other as they cross the membrane (i.e., they are not independent). Therefore, the total electrochemical driving force for GLT1 is the sum of the linked contributions from each co-transported ion. Because one thermodynamic reaction cycle for GLT-1 involves coupled translocation of three sodium ions, one proton and one negative glutamate molecule and countertransport of one potassium ion are quantified as

(A.2)
Δμ~GLT-1=3×(ΔΨNa+ΔGNa)+1×(ΔΨK+ΔGK) +1×(ΔΨGlu+ΔGGlu)+1∑(ΔΨH+ΔGH).

In equilibrium, when $\Delta {\tilde{\mu }}_{\text{GLT1}}=0$ and knowing that the K^+^ term must be negative as it is going in the opposite direction, this equation reduces to

(A.3)
Em=−RT(3ZNa+ZH−ZGlu−ZK)F   ×[ln⁡[Glu−]in[Glu−]out+3ln⁡[Na+]in[Na+]out     −ln⁡[K+]in[K+]out+ln⁡[H+]in[H+]out]⇒Em=−RT2F·ln⁡[[Glu−]in[Na+]in3[H+]in[K+]in−1[Glu−]out[Na+]out3[H+]out[K+]out−1]

which defines the reversal potential for the transporter. It should be noted that this latter equation has a very similar form to the Goldman-Hodgkin-Katz equation for ion channels. The only difference is that ion fluxes are coupled unlike the fluxes for ion channels. The calculated driving force for a transporter can be viewed in the same way as the driving force for an ion channel; thus, there will be no net substrate flux when membrane potential is equal to the reversal potential [12, 13]. This last equation can be rearranged also like this, clearly showing the substrate gradient produced by the transporter:

(A.4)
[Glu−]out[Glu−]in=[Na+]in3[H+]in[K+]in−1[Na+]out3[H+]out[K+]out−1exp−Em(2F/RT).

### B. Derivation of Michaelis-Menten Equation for the Transporter

Kinetic concept takes into account only steady-state velocities of transport that can be divided in adhesion, transport, and release of substrate on other side of the membrane. Let the transporter *T* and the substrate *S* first form the complex *ST* in the outer membrane, and then substrate is transported to the inner membrane and released. The reversal is not taken into consideration. This can be written as follows:

(B.1)
$$\begin{array}{c} T+S_{\text{out}}\overset{k_{1}}{\underset{k_{-1}}{\Leftrightarrow }}TS\overset{k_{2}}{\underset{k_{-2}}{\Leftrightarrow }}T+S_{\text{in}}. \end{array}$$

Formation of *TS* complex is proportional to the concentration of substrate and the free transporter:

(B.2)
$$\begin{array}{c} \text{Formation}=k_{1}\cdot T\cdot S, \end{array}$$

when the complex transports the substrate and releases it inside the cell proportionally to the concentration of *TS*, and also some *TS* complex just releases substrate again without transporting it. Thus, the removal of *TS* from the system is

(B.3)
$$\begin{array}{c} \text{Removal}=k_{-1}\cdot TS+k_{2}\cdot TS. \end{array}$$

After *TS* complex formation the quantity of free transporter left is

(B.4)
$$\begin{array}{c} T=T_{\text{total}}-TS. \end{array}$$

At steady state (at equilibrium), formation and removal of *TS* became the same:

(B.5)
k1·T·Sout =k−1·TS+k2·TS

(B.6)
 ⇒k1·(Ttotal−TS)·Sout=(k−1+k2)·TS

(B.7)
 ⇒k1·Ttotal·Sout=(k−1+k2−k1·Sout)·TS

(B.8)
⇒TS=k1·Ttotal·Soutk−1+k2−k1·Sout⇒Ttotal·Sout((k−1+k2)/k1)+Sout.

As velocity of substrate transport is proportional to *k*_2_ and *TS* : *υ* = *k*_2_ · *TS*_out_, we can write

(B.9)
$$\begin{array}{c} \upsilon =\frac{k_{2}\cdot T_{\text{total}}\cdot S_{\text{out}}}{\left({k_{-}}_{1}+k_{2}\right)/k_{1}+S_{\text{out}}}. \end{array}$$

As in abundance of the substrate all velocity depends only on transporter *T*_total_, maximal velocity of the transport (*V*_max⁡_) is *k*_2_∗*T*_total_ and the constant (*k*_−1_ + *k*_2_)/*k*_1_ = *K*_*m*_, we can write

(B.10)
$$\begin{array}{c} \upsilon =\frac{V_{\max}\cdot S_{\text{out}}}{K_{m}+S_{\text{out}}}. \end{array}$$

The formula allows determination of *K*_*m*_ from the experimental curve: *K*_*m*_ + *S*_out_ = ((*V*_max⁡_ · *S*_out_)/*υ*)⇒*K*_*m*_ = ((*V*_max⁡_ · *S*_out_)/*υ*) − *S*_out_ = *S*_out_((*V*_max⁡_/*υ*) − 1), which means *K*_*m*_ becomes equal to *S*_out_ if ((*V*_max⁡_/*υ*) − 1) = 1, and it happens when *υ* = (*V*_max⁡_/2).

So, when the speed of transport is saturated and becomes *V*_max⁡_ it is simple to find half of *V*_max⁡_ that is equal to *K*_*m*_. Michaelis constant *K*_*m*_ = (*k*_−1_ + *k*_2_)/*k*_1_ are used as a measure of substrate affinity to the transporter. Please, note that *K*_*m*_ does not include the *K*_−2_ constant, reflecting unidirectionality of transporters in the Michaelis-Menten approach.

*Competitive Inhibition from the Michaelis-Menten Point of View.* Transporters can have another substrate *I*. It can bind to the transporter, whether it is transported or not

(B.11)
$$\begin{array}{c} T+I\Leftrightarrow TI. \end{array}$$

The constant of dissociation of this complex can be written as follows:

(B.12)
$$\begin{array}{c} K_{\text{diss}}=K_{i}=\frac{T\cdot I}{TI}\Rightarrow TI=\frac{T\cdot I}{K_{i}}. \end{array}$$

On the other hand, from the equation

(B.13)
d[TS]dt=T·Sout·k1−TS·(k−1+k2)=0⇒T·SoutTS=k−1+k2k1=Km⇒T=KmSout·TS,

if we replace *T* from (B.7) with the last formula, we will get

(B.14)
$$\begin{array}{c} TI=\frac{T\cdot I}{K_{i}}\Rightarrow TI=\frac{K_{m}}{S_{\text{out}}}\cdot \frac{I}{K_{i}}TS. \end{array}$$

The transporter can be in free form or can be occupied by inhibitor or by substrate; therefore, one can write

(B.15)
Ttotal=T+TI+TS,Ttotal=KmSout·TS+KmSout·IKiTS+TS⇒TS=Ttotal·SoutKm(1+I/Ki)+Sout.

This means the velocity of the transport of the main substrate *S* will be

(B.16)
υ=k2·TS=k2·Ttotal·SoutKm(1+I/Ki)+Sout=Vm·SoutKm·α+Sout.

### C. Kinetic Scheme with Two-Substrate Uptake-Release by the Same Transporter

In a general case, the simplest representation of the two-substrate scheme can be determined by set of elementary steps, characterized by their kinetic coefficients:

1. *S*_1_ + *T* → (*S*_1_ *T*) : *k*_11_; —formation of the intermediate complex “first substrate-transporter” (*S*_1_ *T*) outside the cell;
2. (*S*_1_ *T*) → *S*_1_ + *T* : *k*_−11_— (*S*_1_ *T*) dissociation outside the cell;
3. (*S*_1_ *T*) → *S*_1_′ + *T* : *k*_12_—dissociation of (*S*_1_ *T*) complex inside of cell, with substrate *S*_1_′ released inside;
4. *S*_1_′ + *T* → (*S*_1_ *T*) : *k*_−12_—formation of the first substrate complex with transporter inside the cell;
5. *S*_2_ + *T* → (*S*_2_ *T*) : *k*_21_—formation of the intermediate complex “second substrate-transporter” (*S*_2_ *T*) outside the cell;
6. (*S*_2_ *T*) → *S*_2_ + *T* : *k*_−21_—dissociation of (*S*_2_ *T*) complex outside of cell;
7. (*S*_2_ *T*) → *S*_2_′ + *T* : *k*_22_—dissociation of (*S*_2_ *T*) complex inside of cell;
8. *S*_2_′ + *T* → (*S*_2_ *T*) : *k*_−22_—formation of the second substrate complex with transporter inside the cell.

Let us introduce notations of

(C.1)
$$\begin{array}{c} \left[S_{1}\right]=x_{1},\;\;\left[T\right]=y,\\ \left[S_{1}T\right]=z_{1},\;\;\left[S_{1}^{\prime }\right]=s_{1},\\ \left[S_{2}\right]=x_{2},\;\;\left[S_{2}T\right]=z_{2},\\ \left[S_{2}^{\prime }\right]=s_{2}. \end{array}$$

Series of kinetics equations describing dynamics of the system of interest can be represented as follows:

(C.2)
$$\begin{array}{c} \frac{dx_{1}}{dt}=-k_{11}x_{1}y+k_{-11}z_{1}, \end{array}$$

(C.3)
dydt=−k11x1y+k−11z1+k12z1 −k−12ys1−k21x2y+k−21z2 +k22z2−k−22ys2,

(C.4)
$$\begin{array}{c} \frac{dz_{1}}{dt}=k_{11}x_{1}y-k_{-11}z_{1}-k_{12}z_{1}+k_{-12}ys_{1}, \end{array}$$

(C.5)
$$\begin{array}{c} \frac{ds_{1}}{dt}=k_{12}z_{1}-k_{-12}ys_{1}, \end{array}$$

(C.6)
$$\begin{array}{c} \frac{dx_{2}}{dt}=-k_{21}x_{2}y+k_{-21}z_{2}, \end{array}$$

(C.7)
$$\begin{array}{c} \frac{dz_{2}}{dt}=k_{21}x_{2}y-k_{-21}z_{2}-k_{22}z_{2}+k_{-22}ys_{2}, \end{array}$$

(C.8)
$$\begin{array}{c} \frac{ds_{2}}{dt}=k_{22}z_{2}-k_{-22}ys_{2}. \end{array}$$

Summarizing (C.3), (C.4), and (C.7), we will obtain

(C.9)
$$\begin{array}{c} \frac{d\left(y+z_{1}+z_{2}\right)}{dt}=0,\\ y+z_{1}+z_{2}=\text{const}.=T_{0},\\ y=T_{0}-z_{1}-z_{2}. \end{array}$$

As well as we can write

(C.10)
S10=x1+z1+s1,s1=S10−x1−z1,S20=x2+z2+s2,s2=S20−x2−z2.

Therefore, the equation system represented above can be rewritten as follows:

(C.11)
dx1dt=−k11x1T0+(k11x1+k−11)z1+k11x1z2,dz1dt=k11x1(T0−z1−z2)−k−11z1−k12z1 +k−12(T0−z1−z2)(S10−x1−z1),dx2dt=−k21x2T0+(k21x1+k−21)z2+k21x2z1,dz2dt=k21x2(T0−z1−z2)−k−21z2−k22z2 +k−22(T0−z1−z2)(S20−x2−z2).

#### C.1. Equilibrium Conditions for Both Substrates

In equilibrium conditions, we can write

(C.12)
$$\begin{array}{c} K_{11}=\frac{z_{1}}{x_{1}y}=\frac{z_{1}}{x_{1}\left(T_{0}-z_{1}-z_{2}\right)},\\ K_{12}=\frac{ys_{1}}{z_{1}}=\frac{\left(T_{0}-z_{1}-z_{2}\right)\left(S_{10}-x_{1}-z_{1}\right)}{z_{1}},\\ K_{21}=\frac{z_{2}}{x_{2}y}=\frac{z_{2}}{x_{2}\left(T_{0}-z_{1}-z_{2}\right)},\\ K_{22}=\frac{ys_{2}}{z_{2}}=\frac{\left(T_{0}-z_{1}-z_{2}\right)\left(S_{20}-x_{2}-z_{2}\right)}{z_{2}}, \end{array}$$

where

(C.13)
$$\begin{array}{c} K_{11}=\frac{k_{11}}{k_{-11}},\;\;K_{12}=\frac{k_{12}}{k_{-12}},\\ K_{21}=\frac{k_{21}}{k_{-21}},\;\;K_{22}=\frac{k_{22}}{k_{-22}} \end{array}$$

are equilibrium constants of processes of interest. Taking into account relations (C.12) for equilibrium constants, we can form combinations of such constants, which may be represented as follows:

(C.14)
$$\begin{array}{c} K_{11}K_{12}=\frac{S_{10}-x_{1}-z_{1}}{x_{1}},\\ K_{21}K_{22}=\frac{S_{20}-x_{2}-z_{2}}{x_{2}}. \end{array}$$

Let us consider the case, when *T*_0_ ≫ *y*, - all transporter is coupled to complexes of (*S*_1_ *T*) and (*S*_2_ *T*). In this case, we can write with a fair approximation the relations of

(C.15)
$$\begin{array}{c} z_{1}=\left[T_{0}\right]-z_{2},\\ K_{11}K_{12}=\frac{S_{10}-x_{1}-\left[T_{0}\right]+z_{2}}{x_{1}},\\ K_{11}K_{12}x_{1}=S_{10}-x_{1}-\left[T_{0}\right]+z_{2}. \end{array}$$

From the last relationship, the *z*_2_ concentration can be represented as follows:

(C.16)
$$\begin{array}{c} z_{2}=K_{11}K_{12}x_{1}-S_{10}+x_{1}+\left[T_{0}\right]. \end{array}$$

Applying the same approach to equations related to the second substrate and taking into account the last relationship for concentration of *z*_2_ we can write

(C.17)
K21K22=S20−x2−(K11K12x1−S10+x1+[T0])x2=S20−x2−K11K12x1+S10−x1−[T0]x2,x2=S20−K11K12x1+S10−x1−[T0]1+K21K22.

If relationship of

(C.18)
$$\begin{array}{c} S_{10}+S_{20}\gg \left[T_{0}\right] \end{array}$$

is satisfied, we can write

(C.19)
$$\begin{array}{c} x_{2}=\frac{S_{20}+S_{10}}{1+K_{21}K_{22}}\;-\frac{1+K_{11}K_{12}}{1+K_{21}K_{22}}x_{1}, \end{array}$$

and finally we can write

(C.20)
$$\begin{array}{c} \left(1+K_{11}K_{12}\right)x_{1}+\left(1+K_{21}K_{22}\right)x_{2}=S_{10}+S_{20},\\ \alpha x_{1}+\beta x_{2}=S_{10}+S_{20}, \end{array}$$

where

(C.21)
$$\begin{array}{c} \alpha =\left(1+K_{11}K_{12}\right),\\ \beta =\left(1+K_{21}K_{22}\right). \end{array}$$

Let us consider an additional relationship, which can be formed by multiplication of all equilibrium constants, that is, by multiplication of relationships of

(C.22)
$$\begin{array}{c} K_{11}K_{22}=\frac{\left(S_{20}-x_{2}-z_{2}\right)z_{1}}{x_{1}z_{2}},\\ K_{12}K_{21}=\frac{z_{2}\left(S_{10}-x_{1}-z_{1}\right)}{x_{2}z_{1}}. \end{array}$$

In this case, we will obtain relationship of

(C.23)
K11K22K12K21=(S20−x2−z2)(S10−x1−z1)x1x2≈(S20−x2)(S10−x1)x1x2,K11K22K12K21x1x2≈(S20−x2)(S10−x1),

where with respect to relationship (C.18), we may assume that the relationships of

(C.24a)
$$\begin{array}{c} S_{10}\gg z_{1},\\ S_{20}\gg z_{2} \end{array}$$

are satisfied, and we may finally write

(C.24b)
$$\begin{array}{c} \left(K_{11}K_{22}K_{12}K_{21}-1\right)x_{1}x_{2}\approx S_{10}S_{20}-S_{20}x_{1}-S_{10}x_{2}. \end{array}$$

Taking into account relationship of

(C.25)
$$\begin{array}{c} \alpha x_{1}+\beta x_{2}=S_{10}+S_{20}, \end{array}$$

we can write

(C.26)
$$\begin{array}{c} x_{2}=\frac{S_{10}+S_{20}-\alpha x_{1}}{\beta }, \end{array}$$

and if we will substitute the last value to relationship (C.24b), we will obtain

(C.27)
γx1S10+S20−αx1β  ≈S10S20−S20x1−S10S10+S20−αx1β   −αγβx12+γβ(S10+S20)x1+S20x1−S10αβx1   −S10S20+S10β(S10+S20)=0,x12−[S10+S20α+βαγS20−S10γ]x1+βαγS10S20−S10αγ(S10+S20)=0,

(C.28)
$$\begin{array}{c} x_{1}^{2}-ax_{1}+b=0,\\ x_{1}=\frac{a}{2}\pm \sqrt{\frac{a^{2}}{4}-b}, \end{array}$$

where

(C.29)
$$\begin{array}{c} \gamma =\left(K_{11}K_{22}K_{12}K_{21}-1\right),\\ a=\left[\frac{S_{10}+S_{20}}{\alpha }+\frac{\beta }{\alpha \gamma }S_{20}-\frac{S_{10}}{\gamma }\right],\\ b=\frac{\beta }{\alpha \gamma }S_{10}S_{20}-\frac{S_{10}}{\alpha \gamma }\left(S_{10}+S_{20}\right). \end{array}$$

Thus,

(C.30)
x1=12[S10+S20α+βαγS20−S10γ]  ±(14[S10+S20α+βαγS20−S10γ]2    −(βαγS10S20−S10αγ(S10+S20)))1/2 =12[S10(1α−1γ)+S20α(1+βγ)]  ±(14[S10(1α−1γ)+S20α(1+βγ)]2    +1αγ(S10−(β−1)S20)2−S202αγ(β−1)2)1/2,x1=12[a1S10+a2S20] ±(14[a1S10+a2S20]2   + a3(S10−a4S20)2−a3a42S202)1/2,a1=(1α−1γ),a2=(1+βγ),a3=1αγ,a4=(β−1),x2=S10+S20β−αβ ×{12[S10+S20α+βαγS20−S10γ]   ±(14[S10+S20α+βαγS20−S10γ]2     −(βαγS10S20−S10αγ(S10+S20)))1/2},x2=S10+S20β−αβ ×{12[a1S10+a2S20]    ±14[a1S10+a2S20]2+a3(S10−a4S20)2−a3a42S202}.

Solution is physically reasonable, if the relation of

(C.31)
14[S10+S20α+βαγS20−S10γ]2  −(βαγS10S20−S10αγ(S10+S20))≥0

is satisfied. Since parameter *γ* = (*K*_11_ *K*_22_ *K*_12_ *K*_21_ − 1), it can easy be shown that condition (C.31) is satisfied at all reasonable values of *α* and *β* parameters. It also follows from relationships (C.30) that at fixed concentration of *S*_10_ and variable concentration of *S*_20_, equilibrium concentration of *x*_1_ increases with increase of *S*_20_ (effect of *S*_1_ substrate realizing from the cell) and decreasing of equilibrium concentration of *x*_1_ with decreasing of *S*_20_ (effect of *S*_1_ substrate transport inside of the cell). The same behavior follows from relationships of interest for equilibrium concentration of *x*_2_ at fixed concentration of *S*_2_ and variable concentration of *S*_10_. The respective dependences are shown in Figure 1.

*Conclusions of Appendix C.1*

(1) Effect of substrate realizing in case of transportation competition for two substrate system can be observable, if in equilibrium condition most of the transporter is coupled by both substrates of interest: *T*_0_ ≫ *y*.
(2) Efficiency of substrate realizing process is dependent on equilibrium constant values describing processes of substrate-transporter intermediate complex formation.
(3) Correct sign in front of square root in relations (C.30) is defined by the conditions of
  (C.32)
  $$\begin{array}{c} S_{10}\geq x_{1}\geq 0,\\ S_{20}\geq x_{2}\geq 0. \end{array}$$
(4) Relationships (C.30) can be used for analysis of equilibrium substrate concentration dependence on initial substrate concentrations.
(5) The relationship of
  (C.33)
  $$\begin{array}{c} x_{2}=\frac{S_{10}+S_{20}-\alpha x_{1}}{\beta } \end{array}$$
  can be used to determine parameters of *α* and *β*, if concentration of *x*_1_ and *x*_2_ can simultaneously be measured as function of *S*_10_ and *S*_20_ initial concentrations of first and second substrates.

*Procedure of Experimental Measurements of x*_2_ = (*S*_10_ + *S*_20_ − *αx*_1_)/*β Relationship *

(1) Measurements of equilibrium concentrations set of *x*_1_, *x*_2_ versus *S*_10_ and *S*_20_.
  (i) Let us choose a fixed set of concentrations *S*_10,1_ from the interval [*S*_10_^(0)^, *S*_10_^(1)^]. For a fixed concentration *S*_10,1_, let us measure the dependence of concentrations *x*_1_ and *x*_2_ versus [*S*_20_]. The same procedure must be repeated for another fixed concentration *S*_10,2_, the third fixed concentration *S*_10,3_, and so forth.
  (ii) Using the equation
    (C.34)
    $$\begin{array}{c} x_{2}=\frac{S_{10}+S_{20}-\alpha x_{1}}{\beta }=\frac{S_{10}+S_{20}}{\beta }-\frac{\alpha x_{1}}{\beta }, \end{array}$$

it can be rewritten as

(C.35)
$$\begin{array}{c} S_{10}+S_{20}=\alpha x_{1}+\beta x_{2},\\ U=\alpha x_{1}+\beta x_{2}. \end{array}$$

We can experimentally measure {*U*_*i*_}, {*x*_1*i*_}, and {*x*_2*i*_}, with unknown parameters: *α*, *β*. Applying the least-squares method, we can write

(C.36)
$$\begin{array}{c} \frac{\partial }{\partial p}\sum_{i}{\left(U_{i}-\alpha x_{1i}-\beta x_{2i}\right)}^{2}=0\;p=\alpha ,\beta . \end{array}$$

Thus,

(C.37)
∑i(Ui−αx1i−βx2i)x1i=0,∑i(Ui−αx1i−βx2i)x2i=0,∑i(Ui−αx1i−βx2i)x1i  =∑iUix1i−α∑ix1i2−β∑ix2ix1i=0,∑i(Ui−αx1i−βx2i)x2i  =∑iUix2i−α∑ix1ix2i−β∑ix2i2=0.

Main system determinants can be represented as follows:

(C.38)
Δ=|∑ix1i2∑ix2ix1i∑ix2ix1i∑ix2i2|=(∑ix1i2)(∑ix2i2)−(∑ix2ix1i)2,Δα=|∑iUix1i∑ix2ix1i∑iUix2i∑ix2i2|=(∑iUix1i)(∑ix2i2) −(∑iUix2i)(∑ix2ix1i),Δβ=|∑ix1i2∑iUix1i∑ix2ix1i∑iUix2i|=(∑ix1i2)(∑iUix2i) −(∑iUix1i)(∑ix2ix1i).

Therefore,

(C.39)
α=ΔαΔ=(∑iUix1i)(∑ix2i2)−(∑iUix2i)(∑ix2ix1i)(∑ix1i2)(∑ix2i2)−(∑ix2ix1i)2,β=ΔβΔ=(∑ix1i2)(∑iUix2i)−(∑iUix1i)(∑ix2ix1i)(∑ix1i2)(∑ix2i2)−(∑ix2ix1i)2.

Earlier we had determined the parameters of interest as

(C.40)
$$\begin{array}{c} \alpha =\left(1+K_{11}K_{12}\right),\\ \beta =\left(1+K_{21}K_{22}\right). \end{array}$$

That is,

(C.41)
$$\begin{array}{c} K_{11}K_{12}=\alpha -1,\\ K_{21}K_{22}=\beta -1. \end{array}$$

As *K*_12_, *K*_21_, *K*_11_, *K*_22_:

(C.42)
$$\begin{array}{c} K_{11}=\frac{k_{11}}{k_{-11}},\\ K_{12}=\frac{k_{12}}{k_{-12}},\\ K_{21}=\frac{k_{21}}{k_{-21}},\\ K_{22}=\frac{k_{22}}{k_{-22}}, \end{array}$$

we can write

(C.43)
$$\begin{array}{c} \frac{k_{11}}{k_{-11}}\cdot \frac{k_{12}}{k_{-12}}=\alpha -1,\\ \frac{k_{21}}{k_{-21}}\cdot \frac{k_{22}}{k_{-22}}=\beta -1. \end{array}$$

#### C.2. Two-Substrate System Dynamics at the Initial Time

In this case we will not take into account processes (4) and (8):

(C.44)
$$\begin{array}{c} S_{1}^{\prime }+T\to \left(S_{1}T\right),\\ S_{2}^{\prime }+T\;\to \;\left(S_{2}T\right). \end{array}$$

In this case, the series of kinetics equations can be represented as follows:

(C.45)
dx1dt=−k11x1y+k−11z1,dydt=−k11x1y+k−11z1+k12z1 −k21x2y+k−21z2+k22z2,dz1dt=k11x1y−k−11z1−k12z1,dx2dt=−k21x2y+k−21z2,dz2dt=k21x2y−k−21z2−k22z2.

For *z*_1_, *z*_2_, we may use quasi-stationary approximation:

(C.46)
$$\begin{array}{c} \frac{dz_{1}}{dt}=k_{11}x_{1}y-k_{-11}z_{1}-k_{12}z_{1}=0,\\ \frac{dz_{2}}{dt}=k_{21}x_{2}y-k_{-21}z_{2}-k_{22}z_{2}=0. \end{array}$$

Thus,

(C.47)
$$\begin{array}{c} z_{1}=\frac{k_{11}x_{1}y}{k_{-11}+k_{12}},\\ z_{2}=\frac{k_{21}x_{2}y}{k_{-21}+k_{22}}. \end{array}$$

Thus,

(C.48)
y=[T0]−z1−z2=[T0]−k11x1yk−11+k12−k21x2yk−21+k22,y=[T0]1+(k11x1)/(k−11+k12)+(k21x2)/(k−21+k22).

For the initial time after the start of all processes, we can assume that *x*_1_ = *S*_10_ and *x*_2_ = *S*_20_; that is,

(C.49)
$$\begin{array}{c} y=\frac{\left[T_{0}\right]}{1+\left(k_{11}S_{10}\right)/\left(k_{-11}+k_{12}\right)+\left(k_{21}S_{20}\right)/\left(k_{-21}+k_{22}\right)}. \end{array}$$

Hence we can write

(C.50)
υx1=dx1dt=−k11x1y+k−11z1=[−k11+k−11k11k−11+k12] ×(S10[T0])×(1+k11S10k−11+k12+k21S20k−21+k22)−1=k11k12k−11+k12 ×(S10[T0])×(1+k11S10k−11+k12+k21S20k−21+k22)−1=k12KM,1−1S10[T0]1+KM,1−1S10+KM,2−1S20=k12S10[T0]KM,1+S10+KM,1KM,2−1S20=k12S10[T0]KM,1+S10+αS20,

(C.51)
υx2=dx2dt=−k21x2y+k−21k21x2yk−21+k22=[−k21+k−21k21k−21+k22]x2y=k21k22k−21+k22 ×(S20[T0])×(1+k11S10k−11+k12+k21S20k−21+k22)−1=k22KM,2−1S20[T0]1+KM,1−1S10+KM,2−1S20=k22S20[T0]KM,2+KM,1−1KM,2S10+S20=k22S20[T0]KM,2+α−1S10+S20,

where

(C.52)
$$\begin{array}{c} K_{1}^{-1}=\frac{k_{11}}{k_{-11}+k_{12}},\\ K_{2}^{-1}=\frac{k_{21}}{k_{-21}+k_{22}},\\ \alpha =\frac{K_{M,1}}{K_{M,2}} \end{array}$$

are Michaelis constants for the first and second substrate, respectively, and *α*—is a factor of transporter inhibition.

*Conclusions for Substrate Dynamics Section*

(1) Equations
  (C.53)
  $$\begin{array}{c} \upsilon_{x1}=\frac{dx_{1}}{dt}=k_{12}\frac{S_{10}\left[T_{0}\right]}{K_{M,1}+S_{10}+\alpha S_{20}}, \end{array}$$
  (C.54)
  $$\begin{array}{c} \upsilon_{x2}=\frac{dx_{2}}{dt}=k_{22}\frac{S_{20}\left[T_{0}\right]}{K_{M,2}+\alpha^{-1}S_{10}+S_{20}} \end{array}$$
  are the analogs to the Michaelis formula for a two-substrate system. If *S*_20_ = 0, we get the Michaelis formula for the first substrate, and if *S*_10_ = 0, we get the formula for the second one.
(2) Constants *k*_12_, *K*_*M*,1_, *k*_22_, *K*_*M*,2_ can be determined experimentally:
  (iii) *S*_10_ ≫ *K*_*M*,1_  and  *K*_*M*,2_ : *S*_10_ ≫ *αS*_20_:
    (C.55)
    $$\begin{array}{c} \upsilon_{1,s}=k_{12}\left[T_{0}\right],\\ \upsilon_{x2}=k_{22}\frac{\alpha S_{20}\left[T_{0}\right]}{S_{10}}, \end{array}$$
  (iv) *S*_10_ ≪ *αS*_20_ : *αS*_20_ ≫ *K*_*M*,1_:
    (C.56)
    υx1′=dx1dt=k12S10[T0]αS20,υ2,s′=k22[T0],υ1,sυ2,s=k12S10[T0]αS20k22αS20[T0]S10=k12k22[T0]2.

#### C.3. Effect of the Equilibrium Reverse Bias for a First Substrate When a Second One Is Added to the System

If previously the equilibrium was established for a first substrate between the outside concentration of the substrate and the inside concentration, the addition of a second substrate will produce a reverse bias (equilibrium shift). To study this, we first can write the equation representing the velocity of second substrate transport:

(C.57)
υ2=dx2dt=−k21S20k21S20+k−21+k22 ×[T0](K11K12+1)K11S10+K11K12+1=−S20S20+KM,2×[T0](K11K12+1)K11S10+K11K12+1,

where

(C.58)
$$\begin{array}{c} K_{M,2}=\frac{k_{-21}+k_{22}}{k_{21}}. \end{array}$$

In the beginning, at initial time, some of the transporter molecules in the outside bind to the second substrate while inside there is still no second substrate. This means the availability of outside transporter for a first substrate becomes reduced. Thus, equilibrium for a first substrate starts to break down; that is, the velocity of first substrate transport to outside (release) becomes bigger than its transport to the inside. Our model allows evaluation of the release of a first substrate with simple approximation.

Unlike that in the Appendix C.1, where all the transport molecules were available for both substrates, let us approximate that the amount of transporter binding the first substrate outside is reduced by the second substrate also binding to it. Thus, using this rough approximation, the available outside transporter is reduced to the amount:

(C.59)
$$\begin{array}{c} y=y_{0}-z_{2}, \end{array}$$

where

(C.60)
$$\begin{array}{c} y_{0}=\frac{\left[T_{0}\right]\left(K_{11}K_{12}+1\right)}{K_{11}S_{10}+K_{11}K_{12}+1},\\ z_{2}=\frac{k_{21}S_{20}y_{0}}{k_{21}S_{20}+k_{-21}+k_{22}}, \end{array}$$

while inside (the cell) the amount of transporter available for the first substrate still remains *y*_0_. Consequently, the velocity of the first substrate reverse flow can be represented as

(C.61)
$$\begin{array}{c} \upsilon_{x1}=\frac{dx_{1}}{dt}=-k_{11}^{\prime }x_{1,e}y+k_{-11}^{\prime }s_{1,e}y_{0}, \end{array}$$

where *k*_11_′ and *k*_−12_′ are some effective parameters that can be only analytically solved in more complex model that will implicate intermediate transporter-substrate complexes and are out of the scope of this study. Here we can only get formulas for the initial times during the start of the process and far from equilibrium for a second transporter. In (C.61) we had balanced the flow disparity between the first substrate in-flow (uptake) and out-flow (release), where *x*_1,*e*_ and *s*_1,*e*_ are the equilibrium concentrations of the first substrate outside and inside (the cell). Thus, these concentrations can be approximately found using the approximation that still the system is close to the equilibrium for a first substrate:

(C.62)
$$\begin{array}{c} x_{1}\Leftrightarrow s_{1},\\ K_{0}=\frac{s_{1,e}}{x_{1,e}}=\frac{S_{10}-x_{1,e}}{x_{1,e}}=\frac{k_{11}^{\prime }}{k_{-11}^{\prime }},\\ x_{1,e}=\frac{S_{10}}{K_{0}+1},\\ s_{1,e}=\frac{K_{0}S_{10}}{K_{0}+1}. \end{array}$$

Thus,

(C.63)
υx1=dx1dt=−k11′x1,ey+k−11′s1,ey0=−k11′S10K0+1y0k−21+k22k21S20+k−21+k22 +k−11′K0S10K0+1y0=S10K0+1y0[k−11′K0−k11′k−21+k22k21S20+k−21+k22]=k11′S10K0+1y0[1−KM,2S20+KM,2].

For initial time conditions with relatively good precision, we can accept that

(C.64)
$$\begin{array}{c} \frac{k_{11}^{\prime }S_{10}}{K_{0}+1}y_{0}\approx A=\text{Const}., \end{array}$$

as

(C.65)
k11′S10K0+1y0=k11′S10K0+1×[T0](K11K12+1)K11S10+K11K12+1=k11′S10[T0]K0+1×1KSS10+1=k11′S10[T0]K0+1×KZ≈A=Const.,

where

(C.66)
KS=K11K11K12+1,KZ=1KSS10+1,υx1=k11′S10[T0]K0+1×KZ[1−KM,2S20+KM,2]=A[1−KM,2S20+KM,2].

Thus

(72∗)
$$\begin{array}{c} \upsilon_{x1}=A\left[1-\frac{K_{M,2}}{S_{20}+K_{M,2}}\right]. \end{array}$$

First substrate reverse flow (the release speed) velocity dependence on the external second substrate concentration is represented on Figure 2.

One can see that the reverse flow of the first substrate is absent if *S*_20_ = 0, because in that case the formula (72∗) gives us zero velocity. When the second substrate is added externally the second term inside square brackets becomes reduced and the velocity augments. Thus, the velocity of reversed transport grows with the second substrate concentration and until a limit:

(C.67)
$$\begin{array}{c} \upsilon_{x1}=\frac{k_{11}^{\prime }S_{10}}{K_{0}+1}y_{0}. \end{array}$$
